# Crystal structure and Hirshfeld surface analysis of two organic salts based on 1,3,4-thia­diazole derivatives

**DOI:** 10.1107/S2056989022012154

**Published:** 2023-01-12

**Authors:** Lidiya Izotova, Gulnara Shakirzyanova, Omankul Xolbekov, Shukhrat Turageldiyev, Bahrom Babaev, Bahtiyar Ibragimov

**Affiliations:** a Institute of Bioorganic Chemistry, UzAS, M.Ulugbek Str., 83, 100125, Tashkent, Uzbekistan; Universidad de Los Andes Mérida, Venezuela

**Keywords:** crystal structure, organic salt, hydrogen bonding, 1,3,4-thia­diazole derivatives

## Abstract

During attempts to achieve inter­action between 2-amino-5-ethyl-1,3,4-thia­diazole with oxalyl chloride and 5-mercapto-3-phenyl-1,3,4-thia­diazol-2-thione with various diacid anhydrides, we obtained two co-crystals (organic salts), namely, 2-amino-5-ethyl-1,3,4-thia­diazol-3-ium hemioxalate, C_4_H_8_N_3_S^+^·0.5C_2_O_4_^2−^, (I), and 4-(di­methyl­amino)­pyridin-1-ium 4-phenyl-5-sulfanyl­idene-4,5-di­hydro-1,3,4-thia­diazole-2-thiol­ate, C_7_H_11_N_2_^+^·C_8_H_5_N_2_S_3_^−^, (II). Both solids were investigated by single-crystal X-ray diffraction and by Hirshfeld surface analysis. An infinite one-dimensional chain along [100] is generated through O—H⋯O inter­actions between the oxalate anion and two 2-amino-5-ethyl-1,3,4-thia­diazol-3-ium cations in compound (I), and a three-dimensional supra­molecular framework is generated through C—H⋯O and π–π inter­actions. In compound (II), an organic salt is formed by a 4-phenyl-5-sulfanyl­idene-4,5-di­hydro-1,3,4-thia­diazole-2-thiol­ate anion and a 4-(di­methyl­amino)­pyridin-1-ium cation, which are combined by an N—H⋯S hydrogen-bonding inter­action, forming a zero-dimensional structural unit. As a result of inter­molecular π–π inter­actions, the structural units are combined into a one-dimensional chain running along the *a-*axis direction.

## Abstract 2

Two organic salts based on 1,3,4 thia­diazole derivatives have been obtained and their structures have been established by single-crystal X-ray analysis.

## 1. Chemical context

In the field of medicinal chemistry, the search for new selective drugs with reduced toxicity is ongoing. Heterocyclic compounds with the 1,3,4-thia­diazole structural unit are very attractive for the production of pharmaceuticals as 1,3,4-thia­diazole derivatives exhibit a wide spectrum of biological activities. The 1,3,4-thia­diazole moiety acts as a hydrogen-binding dominant unit on the one hand and as an electron-donor unit on the other (Sharma *et al.*, 2013 ▸). The sulfur atom of the thia­diazole moiety gives lipophilic properties to these compounds, which provides better permeability through biological membranes (Song *et al.*, 1999 ▸). The thia­diazole nucleus with its N–C–S linkage exhibits a large number of biological activities (Kurtzer *et al.*, 1965 ▸). It has been found that derivatives of 1,3,4-thia­diazole have diverse pharmacological activities such as fungicidal, insecticidal, bactericidal, herbicidal, anti-tumor (Shivarama Holla *et al.*, 2002 ▸), anti-inflammatory and anti­viral (Witkoaski *et al.*,1972 ▸). A number of 1,3,4-thia­diazo­les exhibit anti­bacterial properties similar to those of well-known sulfonamide drugs. 1,3,4-Thia­diazole derivatives have been patented for agricultural use, as herbicides and bactericides. According to these findings and in a continuation of our work on synthesizing various condensed-bridge bioactive mol­ecules bearing multifunctional and pharmaceutically active groups (Priya *et al.*, 2005 ▸; Sadashiva *et al.*, 2004 ▸), we have investigated the structural properties of two new 1,3,4-thia­diazole derivatives. 
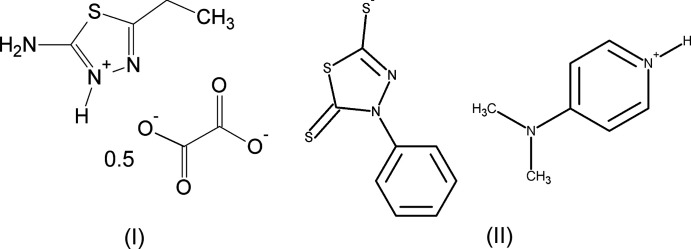


## 2. Structural commentary

**Figure 1:**
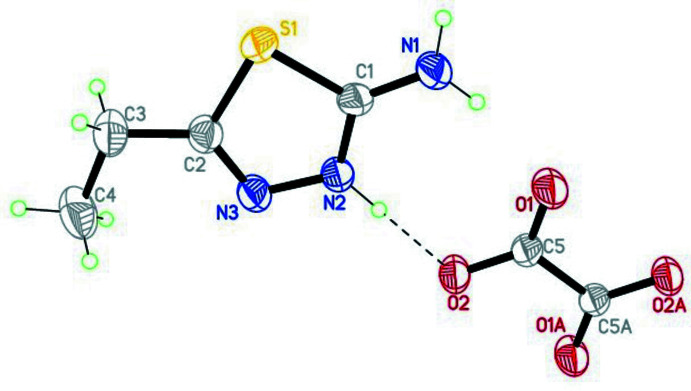
The mol­ecular structure of compound (I), with the atom labeling and displacement ellipsoids drawn at the 40% probability level. The dashed line represents the intra­molecular hydrogen bond. Symmetry code: (A) 2 − *x*, 1 − *y*, 1 − *z*.

**Figure 2:**
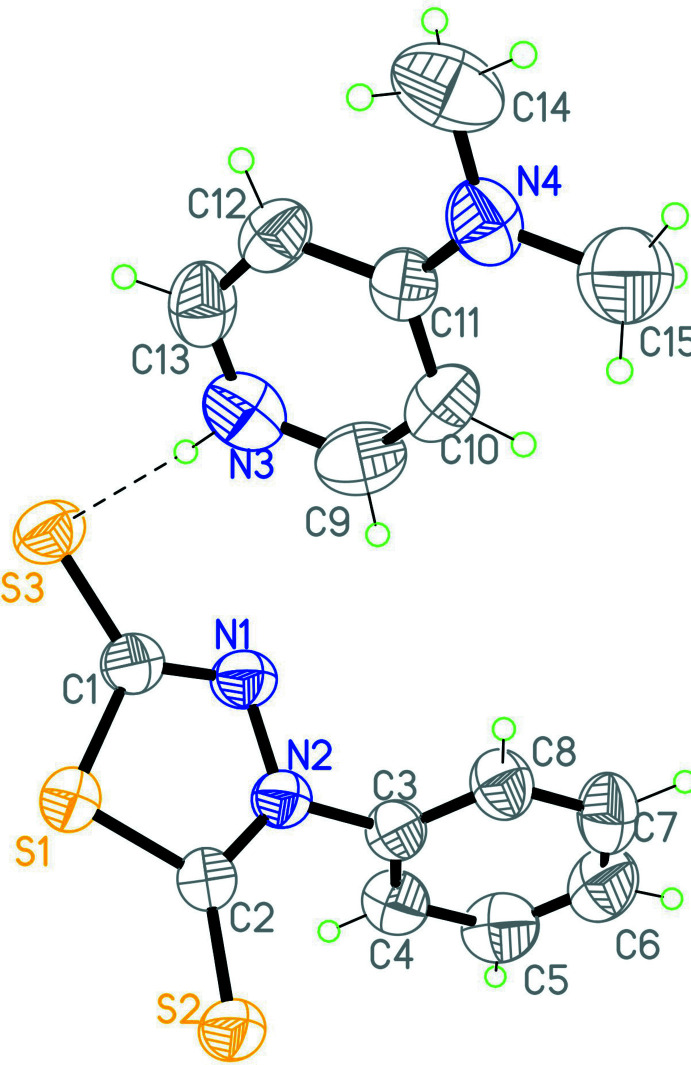
The mol­ecular structure of compound (II), with the atom labeling and displacement ellipsoids drawn at the 40% probability level. The dashed line represents the intra­molecular hydrogen bond.

The mol­ecular structure of compound (I) is illustrated in Fig. 1 ▸. The compound consists of two nearly flat 2-amino-5-ethyl-1,3,4-thia­diazol-3-ium cations and an oxalate anion. The ethyl unit of the 2-amino-5-ethyl-1,3,4-thia­diazol-3-ium cation has an extended conformation and is almost in the same plane as the thia­diazole ring, as indicated by the torsion angle S1—C2—C3—C4 = −176.16 (15)°. The oxalate anion is also in the plane of the cation [the angle between the root-mean-square planes of these mol­ecules is 5.71 (2)°]. The mol­ecular structure of compound (II) is illustrated in Fig. 2 ▸. In the 4-phenyl-5-sulfanyl­idene-4,5-di­hydro-1,3,4-thia­diazole-2-thiol­ate moiety, the phenyl ring is inclined by 69.08 (14)° to the plane of the thia­diazole ring. The 4-(di­methyl­amino)­pyridin-1-ium is almost planar, the largest deviation from the root-mean-square plane of the mol­ecule being 0.01 Å.

## 3. Supra­molecular features

**Figure 3:**
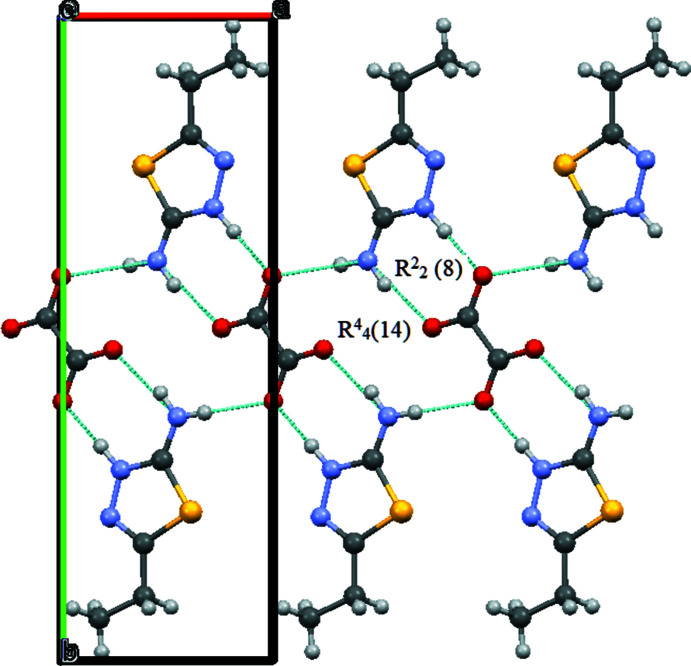
Packing diagram of compound (I) viewed down the *c*–axis. Hydrogen bonds are shown as dashed lines.

**Figure 4:**
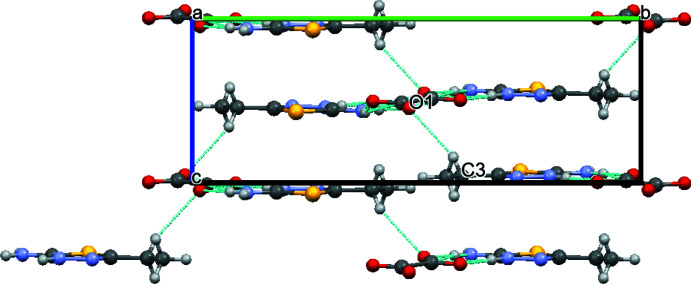
Packing diagram of compound (I) viewed down the *a*-axis. Hydrogen bonds are shown as dashed lines.

In the asymmetric unit of compound (I) there is a protonated 2-amino-5-ethyl-1,3,4-thia­diazole mol­ecule (cation) and half of a doubly deprotonated oxalic acid mol­ecule (anion) (it is on a special position: there is a center of inversion in the middle of the mol­ecule), *i.e.* the mol­ecular ratio is 2:1. The oxygen atoms of the oxalate anion are involved in inter­molecular hydrogen bonding (Table 1 ▸) with neighboring cationic species, leading to the formation of one-dimensional infinite chains. Such chains are packed parallel to each other in the [100] direction in the crystal structure (Fig. 3 ▸). Each chain consists of alternate eight- and fourteen-membered (including hydrogen atoms) conjugated rings, with the graph-set notations 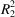 (8) and 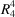 (14), respectively, according to the hydrogen-bonding patterns defined by Etter *et al.* (1993 ▸). These chains are inter­connected *via* C—H⋯O (Table 1 ▸, Fig. 4 ▸) and π–π inter­actions [*Cg*1⋯*Cg*1( 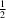 + *x*, 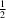 − *y*, 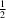 + *z*) = 3.7734 (10) Å, where *Cg*1 is the centroid of the S1/C1/N2/N3/C2 ring].

**Figure 5:**
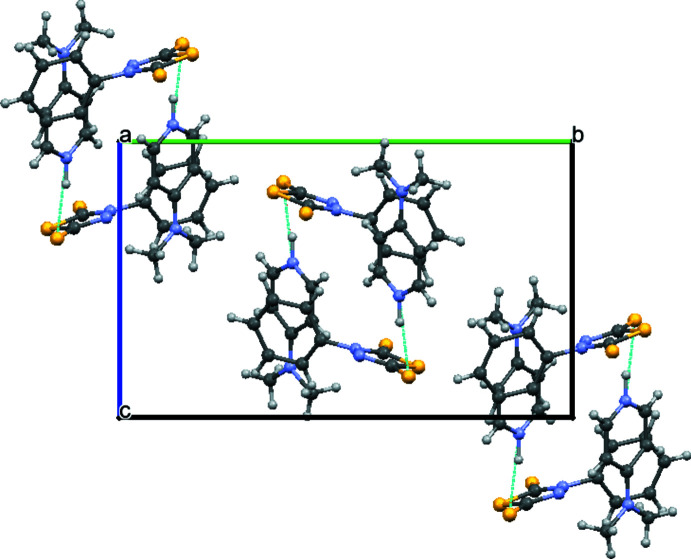
Packing diagram of compound (II) viewed down the *a*-axis. The hydrogen bonds are shown as dashed lines.

In compound (II), the asymmetric unit contains a 4-(di­methyl­amino)­pyridin-1-ium cation and a 4-phenyl-5-sulfanyl­idene-4,5-di­hydro-1,3,4-thia­diazole-2-thiol­ate anion, *i.e*. the mol­ecular ratio is 1:1. The cation and anion are combined by an N—H⋯S hydrogen-bonding inter­action (Table 2 ▸) and form 0-D structural units. As a result of inter­molecular π–π inter­actions between the benzene rings of two equivalent anions of the 4-(di­methyl­amino)­pyridin-1-ium unit [*Cg*1⋯*Cg*1(1 − *x*, 1 − *y*, 1 − *z*) = 4.311 (2) Å, *Cg*1 is the centroid of the C3–C8 ring], the structural units combine as a building block of a one-dimensional chain running along the *a-*axis direction (Fig. 5 ▸).

## 4. Database survey

A search of the Cambridge Structural Database (Version 5.41, September 2021; Groom *et al.*, 2016 ▸) revealed that there are two structures of organic salts containing the compounds mentioned in this article. The first structure is that of 2-amino-5-ethyl-1,3,4-thia­diazole with 2-4 di­chloro­phen­oxy acetic acid (XAPXIV; Lynch *et al.*, 1999 ▸). This structure is considered among proton-transfer complexes and the dominant inter­molecular association is an 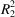 (8) graph-set dimer across the N3*A*/N21*A* site to the two carboxyl­ate oxygen atoms. The second structure is for bis­(4-amino­pyridine-*N*)tri­methyl­tin with 3-phenyl-1,3,4-thia­diazo­line-2-thione-5-thiol­ate (XIGPEI; [Berceanc *et al.*, 2002 ▸). In this complex, the 4-*N*-amino­pyridine, being coordinatively bound to the tin atom, participates in a weak hydrogen bond [N—H⋯S = 3.366 (2) Å, 159°] with the 1,3,4-thia­diazole mol­ecule.

## 5. Hirshfeld surface calculation

**Figure 6:**
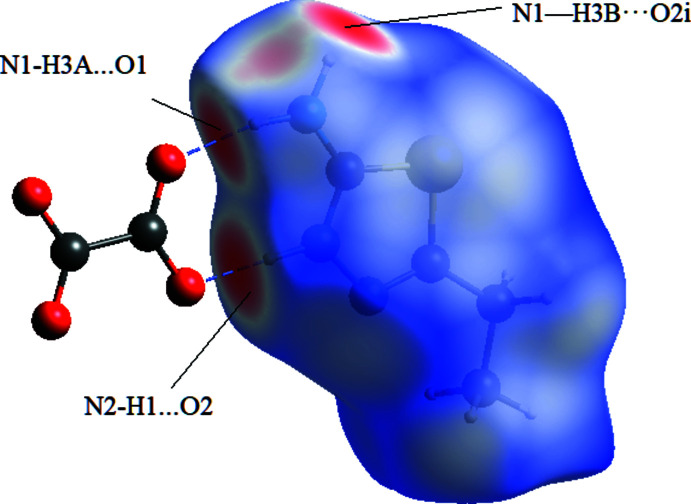
The Hirshfeld surface mapped over *d*_norm_ for compound (I) indicates that the most important contributions to the crystal packing are from O⋯H/H⋯O (39.1%) and H⋯H (29.0%) inter­actions.

**Figure 7:**
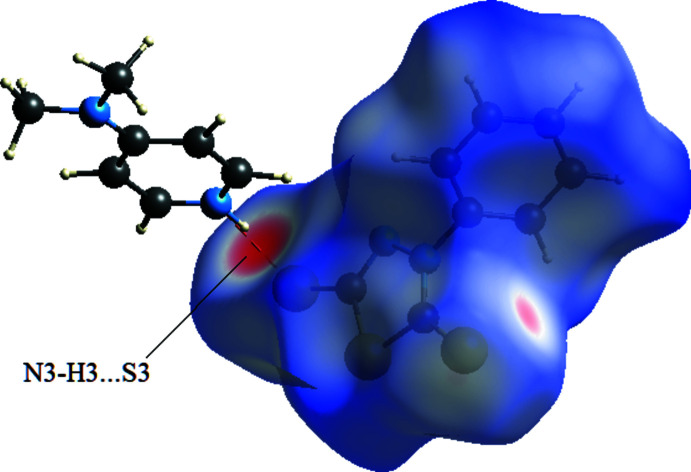
The Hirshfeld surface mapped over *d*_norm_ for compound (II) indicates that the most important contributions to the crystal packing are from S⋯H/H⋯S (35.3%), H⋯H (31.5%) and C⋯H/H⋯C (20.3%) inter­actions.

**Figure 8:**
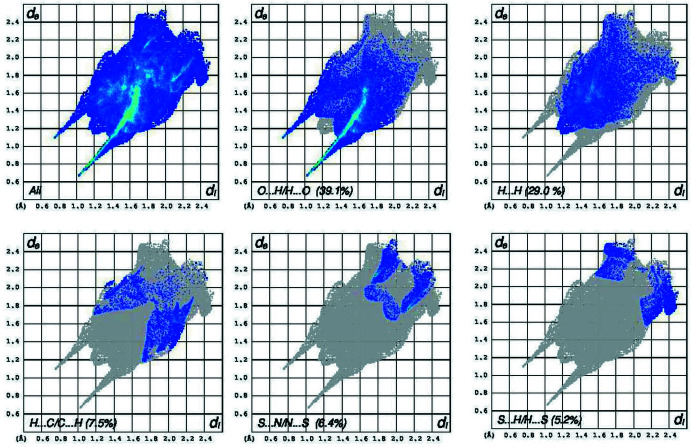
The two-dimensional fingerprint plots for compound (I). The *d*_i_ and *d*_e_ values are the closest inter­nal and external distances (in Å) from a given point on the Hirshfeld surface depicted in Fig. 6 ▸.

**Figure 9:**
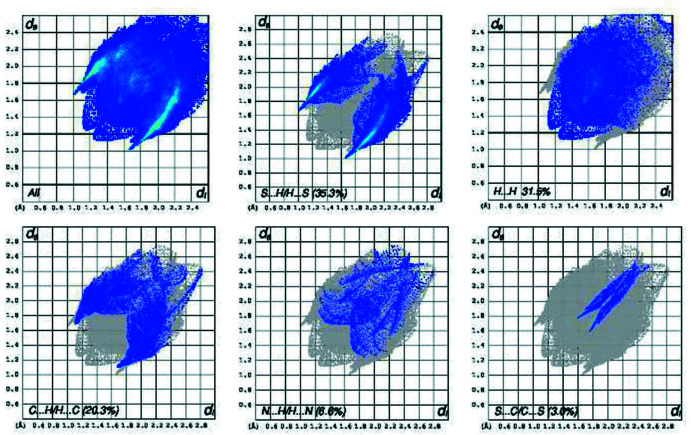
The two-dimensional fingerprint plots for compound (II). The *d*_i_ and *d*_e_ values are the closest inter­nal and external distances (in Å) from a given point on the Hirshfeld surface depicted in Fig. 7 ▸.

In order to visualize the inter­molecular inter­actions in the structures of compounds (I) and (II), a Hirshfeld surface analysis was carried out using *CrystalExplorer 17.5* (Turner *et al.*, 2017 ▸). The Hirshfeld surface mapped over *d*_norm_ (Fig. 6 ▸) shows that in (I), the expected bright-red spots near atoms O1 and O2, involved in the hydrogen-bonding inter­actions. Fingerprint plots (Fig. 8 ▸) reveal that O⋯H/H⋯O, H⋯H and H⋯C/C⋯H inter­actions make the greatest contributions to the surface contacts, while S⋯N/N⋯S, S⋯H/H⋯S, S⋯S contacts are less significant. In (II), the greatest contributions to the surface contacts are from H⋯S/S⋯H, H⋯H and C⋯H/H⋯C inter­actions, with smaller contributions from N⋯H/H⋯N and C⋯C inter­actions (Fig. 7 ▸, Fig. 9 ▸).

## 6. Synthesis and crystallization

*Synthesis of 2-amino-5-ethyl-1,3,4-thia­diazole:*

Propionic acid (0.108 mol) was mixed with 16 g of sulfuric acid (94%). The reaction temperature was allowed to reach 333–343 K, and then, under the same conditions, 0.1 mol of thio­semicarbazide were added. The mixture was stirred for 3 h at 333–343 K, water and charcoal were added, and the mixture was stirred for 40 minutes. At the end of the reaction, the solution was filtered. Then, 44% sodium hydroxide solution was added to get a solution with pH 9.5–10. After cooling the reaction to 303–308 K, the mixture was filtered. The precipitate was washed with water (303 K) and allowed to dry to give the title compound (12 g, 93%), m.p. 460–467 K. IR (cm^−1^): 3290, 2980, 2780; 1640.

Compound (I) was obtained using the procedure described by Harris *et al.* (1984 ▸). We tried to achieve inter­action between 2-amino-5-ethyl-1,3,4-thia­diazole and oxalyl chloride. For this, 20 mmol oxalyl dichloride were mixed with 40 mmol of 2-amino-5-ethyl-1,3,4-thia­diazole in 15 ml of dry acetone, and stirred under boiling acetone for 10 h. The solvent was then removed by rotary evaporation, and the residue was purified by recristallization from water. Beige block-shaped crystals were obtained after one week of slow evaporation of the solvent. We presume that oxalyl chloride was transformed to oxalic acid upon treatment with water in the last step of the reaction.

Compound (II) was obtained during a typical procedure (Sheikh *et al.*, 2010 ▸) for the etherification reaction between 5-mercapto-3-phenyl-1,3,4-thia­diazol-2-thione and glutaric anhydride. The isolated reaction products were amorphous. For purification, the reaction products were treated by filtration in ethyl alcohol. Colorless needle-like single crystals were afforded after 2 days by slow evaporation of the solvent.

## 7. Refinement

Crystal data, data collection and structure refinement details are summarized in Table 3 ▸. In (I), atom H1 (at protonated atom N2 of 2-amino-5-ethyl-1,3,4-thia­diazol-3-ium) was located from difference-Fourier maps. All other H atoms were placed in idealized positions (N—H = 0.86, C—H = 0.96–0.97 Å) and refined as riding on their carrier atoms [*U*_iso_(H) = 1.2*U*_eq_(C,N) or 1.5*U*_eq_(C-meth­yl)]. In (II), all hydrogen atoms except those of the methyl groups in 4-(di­methyl­amino)­pyridin-1-ium were located from difference Fourier-maps and freely refined. Methyl H atoms were positioned geometrically and refined as riding [C—H = 0.96 Å; *U*_iso_(H) = 1.5*U*_eq_(C)].

## Supplementary Material

Crystal structure: contains datablock(s) I, II, global. DOI: 10.1107/S2056989022012154/dj2057sup1.cif

Structure factors: contains datablock(s) I. DOI: 10.1107/S2056989022012154/dj2057Isup2.hkl

Structure factors: contains datablock(s) II. DOI: 10.1107/S2056989022012154/dj2057IIsup3.hkl

CCDC references: 2232672, 2232671

Additional supporting information:  crystallographic information; 3D view; checkCIF report

## Figures and Tables

**Table 1:** Hydrogen-bond geometry (Å, °) for (I)

*D*—H⋯*A*	*D*—H	H⋯*A*	*D*⋯*A*	*D*—H⋯*A*
N1—H3*A*⋯O1	0.86	1.92	2.765 (2)	167
N1—H3*B*⋯O2^i^	0.86	1.99	2.821 (2)	162
N2—H1⋯O2	0.92 (3)	1.78 (3)	2.6989 (19)	178 (2)
C3—H1*C*⋯O1^ii^	0.97	2.65	3.479 (2)	143

Symmetry codes: (i) 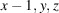 ; (ii) 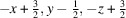 .

**Table 2:** Hydrogen-bond geometry (Å, °) for (II)

*D*—H⋯*A*	*D*—H	H⋯*A*	*D*⋯*A*	*D*—H⋯*A*
N3—H3⋯S3	1.02 (5)	2.16 (5)	3.173 (3)	173 (4)

**Table 3:** Experimental details

	(I)	(II)
Crystal data		
Chemical formula	C_4_H_8_N_3_S^+^·0.5C_2_O_4_^2−^	C_7_H_11_N_2_^+^·C_8_H_5_N_2_S_3_^−^
*M*_r_	174.20	348.50
Crystal system, space group	Monoclinic, *P*2_1_/*n*	Monoclinic, *P*2_1_/*n*
Temperature (K)	293	293
*a*, *b*, *c* (Å)	6.4215 (1), 18.1227 (3), 7.2155 (2)	9.6422 (2), 17.1758 (3), 10.6080 (2)
β (°)	113.095 (3)	99.546 (2)
*V* (Å^3^)	772.41 (3)	1732.49 (6)
*Z*	4	4
Radiation type	Cu *K*α	Cu *K*α
μ (mm^−1^)	3.39	3.92
Crystal size (mm)	0.32 × 0.18 × 0.10	0.20 × 0.17 × 0.12

Data collection		
Diffractometer	XtaLAB Synergy, Single source at home/near, HyPix3000	XtaLAB Synergy, Single source at home/near, HyPix3000
Absorption correction	Multi-scan (*CrysAlis PRO*; Rigaku OD, 2020 ▸)	Multi-scan (*CrysAlis PRO*; Rigaku OD, 2020 ▸)
*T*_min_, *T*_max_	0.131, 1.000	0.123, 1.000
No. of measured, independent and observed [*I* > 2σ(*I*)] reflections	3645, 1480, 1366	16519, 3341, 2681
*R*_int_	0.019	0.047
(sin θ/λ)_max_ (Å^−1^)	0.613	0.615

Refinement		
*R*[*F* ^2^ > 2σ(*F* ^2^)], *wR*(*F* ^2^), *S*	0.035, 0.095, 1.12	0.047, 0.142, 1.09
No. of reflections	1480	3341
No. of parameters	104	234
H-atom treatment	H atoms treated by a mixture of independent and constrained refinement	H atoms treated by a mixture of independent and constrained refinement
Δρ_max_, Δρ_min_ (e Å^−3^)	0.35, −0.33	0.43, −0.48

Computer programs: *CrysAlis PRO* (Rigaku OD, 2020 ▸), *SHELXT2018/2* (Sheldrick, 2015*a* ▸), *SHELXL2018/3* (Sheldrick, 2015*b* ▸), *XP* (Siemens, 1994 ▸), *Mercury* (Macrae *et al.*, 2020 ▸) and *publCIF* (Westrip, 2010 ▸).

## supplementary crystallographic information

### 2-Amino-5-ethyl-1,3,4-thiadiazol-3-ium hemioxalate (I) Crystal data

**Table:**

C_4_H_8_N_3_S^+^·0.5C_2_O_4_^2^^−^	*F*(000) = 364
*M**_r_* = 174.20	*D*_x_ = 1.498 Mg m^−^^3^
Monoclinic, *P*2_1_/*n*	Cu *K*α radiation, λ = 1.54184 Å
*a* = 6.4215 (1) Å	Cell parameters from 2613 reflections
*b* = 18.1227 (3) Å	θ = 4.9–70.9°
*c* = 7.2155 (2) Å	µ = 3.39 mm^−^^1^
β = 113.095 (3)°	*T* = 293 K
*V* = 772.41 (3) Å^3^	Needle, beige
*Z* = 4	0.32 × 0.18 × 0.10 mm

### 2-Amino-5-ethyl-1,3,4-thiadiazol-3-ium hemioxalate (I) Data collection

**Table:**

XtaLAB Synergy, Single source at home/near, HyPix3000 diffractometer	*R*_int_ = 0.019
/ω scans	θ_max_ = 71.1°, θ_min_ = 4.9°
Absorption correction: multi-scan (CrysAlisPro; Rigaku OD, 2020)	*h* = −7→7
*T*_min_ = 0.131, *T*_max_ = 1.000	*k* = −22→11
3645 measured reflections	*l* = −8→8
1480 independent reflections	3 standard reflections every 100 reflections
1366 reflections with *I* > 2σ(*I*)	intensity decay: 2.6%

### 2-Amino-5-ethyl-1,3,4-thiadiazol-3-ium hemioxalate (I) Refinement

**Table:**

Refinement on *F*^2^	Primary atom site location: dual
Least-squares matrix: full	Hydrogen site location: mixed
*R*[*F*^2^ > 2σ(*F*^2^)] = 0.035	H atoms treated by a mixture of independent and constrained refinement
*wR*(*F*^2^) = 0.095	*w* = 1/[σ^2^(*F*_o_^2^) + (0.0493*P*)^2^ + 0.2028*P*] where *P* = (*F*_o_^2^ + 2*F*_c_^2^)/3
*S* = 1.12	(Δ/σ)_max_ < 0.001
1480 reflections	Δρ_max_ = 0.35 e Å^−^^3^
104 parameters	Δρ_min_ = −0.33 e Å^−^^3^
0 restraints	

### 2-Amino-5-ethyl-1,3,4-thiadiazol-3-ium hemioxalate (I) Special details

**Table:**

Geometry. All esds (except the esd in the dihedral angle between two l.s. planes) are estimated using the full covariance matrix. The cell esds are taken into account individually in the estimation of esds in distances, angles and torsion angles; correlations between esds in cell parameters are only used when they are defined by crystal symmetry. An approximate (isotropic) treatment of cell esds is used for estimating esds involving l.s. planes.

### 2-Amino-5-ethyl-1,3,4-thiadiazol-3-ium hemioxalate (I) Fractional atomic coordinates and isotropic or equivalent isotropic displacement parameters (Å^2^)

**Table:**

	*x*	*y*	*z*	*U*_iso_*/*U*_eq_	
S1	0.38930 (7)	0.23126 (2)	0.56878 (7)	0.03711 (17)	
O2	0.9971 (2)	0.40285 (7)	0.5037 (2)	0.0441 (3)	
O1	0.7585 (2)	0.48309 (7)	0.5457 (3)	0.0525 (4)	
N2	0.7208 (2)	0.29656 (8)	0.5395 (2)	0.0355 (3)	
N3	0.7715 (3)	0.22308 (8)	0.5338 (2)	0.0376 (4)	
N1	0.4525 (3)	0.37853 (8)	0.5616 (3)	0.0443 (4)	
H3A	0.532006	0.415948	0.555662	0.053*	
H3B	0.324769	0.385097	0.571813	0.053*	
C5	0.9282 (3)	0.46721 (9)	0.5139 (3)	0.0325 (4)	
C1	0.5255 (3)	0.31189 (9)	0.5552 (3)	0.0327 (4)	
C2	0.6144 (3)	0.18227 (10)	0.5465 (3)	0.0349 (4)	
C3	0.6113 (4)	0.09996 (10)	0.5415 (3)	0.0456 (5)	
H1B	0.482610	0.083694	0.423726	0.055*	
H1C	0.590798	0.081757	0.659693	0.055*	
C4	0.8242 (4)	0.06663 (12)	0.5359 (4)	0.0592 (6)	
H2B	0.812072	0.013808	0.532857	0.089*	
H2C	0.843836	0.083416	0.417603	0.089*	
H2D	0.952085	0.081478	0.653703	0.089*	
H1	0.816 (4)	0.3332 (16)	0.530 (4)	0.065 (7)*	

### 2-Amino-5-ethyl-1,3,4-thiadiazol-3-ium hemioxalate (I) Atomic displacement parameters (Å^2^)

**Table:**

	*U*^11^	*U*^22^	*U*^33^	*U*^12^	*U*^13^	*U*^23^
S1	0.0324 (3)	0.0330 (3)	0.0516 (3)	−0.00368 (16)	0.0226 (2)	0.00117 (17)
O2	0.0398 (7)	0.0253 (6)	0.0797 (9)	−0.0001 (5)	0.0368 (7)	−0.0005 (6)
O1	0.0431 (8)	0.0321 (7)	0.1006 (11)	0.0011 (6)	0.0480 (8)	0.0010 (7)
N2	0.0316 (7)	0.0275 (7)	0.0542 (9)	0.0018 (6)	0.0243 (7)	0.0047 (6)
N3	0.0364 (8)	0.0304 (7)	0.0516 (9)	0.0046 (6)	0.0231 (7)	0.0037 (6)
N1	0.0372 (8)	0.0303 (8)	0.0750 (11)	0.0040 (6)	0.0324 (8)	0.0048 (7)
C5	0.0298 (8)	0.0271 (8)	0.0447 (9)	−0.0014 (7)	0.0191 (7)	−0.0001 (7)
C1	0.0274 (8)	0.0329 (9)	0.0405 (9)	0.0007 (6)	0.0163 (7)	0.0037 (7)
C2	0.0376 (9)	0.0312 (9)	0.0390 (9)	0.0009 (7)	0.0185 (7)	0.0029 (7)
C3	0.0579 (12)	0.0298 (9)	0.0549 (11)	0.0000 (8)	0.0282 (9)	0.0009 (8)
C4	0.0751 (15)	0.0377 (11)	0.0743 (14)	0.0152 (11)	0.0394 (12)	0.0029 (10)

### 2-Amino-5-ethyl-1,3,4-thiadiazol-3-ium hemioxalate (I) Geometric parameters (Å, º)

**Table:**

S1—C1	1.7255 (17)	N1—H3B	0.8600
S1—C2	1.7565 (18)	C5—C5^i^	1.564 (3)
O2—C5	1.260 (2)	C2—C3	1.492 (3)
O1—C5	1.232 (2)	C3—C4	1.510 (3)
N2—C1	1.332 (2)	C3—H1B	0.9700
N2—N3	1.375 (2)	C3—H1C	0.9700
N2—H1	0.92 (3)	C4—H2B	0.9600
N3—C2	1.283 (2)	C4—H2C	0.9600
N1—C1	1.303 (2)	C4—H2D	0.9600
N1—H3A	0.8600		
			
C1—S1—C2	88.23 (8)	N3—C2—S1	114.42 (13)
C1—N2—N3	116.50 (14)	C3—C2—S1	120.25 (14)
C1—N2—H1	122.0 (17)	C2—C3—C4	113.38 (17)
N3—N2—H1	121.5 (17)	C2—C3—H1B	108.9
C2—N3—N2	110.74 (15)	C4—C3—H1B	108.9
C1—N1—H3A	120.0	C2—C3—H1C	108.9
C1—N1—H3B	120.0	C4—C3—H1C	108.9
H3A—N1—H3B	120.0	H1B—C3—H1C	107.7
O1—C5—O2	125.68 (16)	C3—C4—H2B	109.5
O1—C5—C5^i^	117.07 (18)	C3—C4—H2C	109.5
O2—C5—C5^i^	117.25 (18)	H2B—C4—H2C	109.5
N1—C1—N2	124.08 (16)	C3—C4—H2D	109.5
N1—C1—S1	125.82 (13)	H2B—C4—H2D	109.5
N2—C1—S1	110.09 (13)	H2C—C4—H2D	109.5
N3—C2—C3	125.32 (17)		
			
C1—N2—N3—C2	−0.4 (2)	N2—N3—C2—S1	−0.44 (19)
N3—N2—C1—N1	−179.71 (16)	C1—S1—C2—N3	0.89 (14)
N3—N2—C1—S1	1.1 (2)	C1—S1—C2—C3	−178.47 (15)
C2—S1—C1—N1	179.76 (17)	N3—C2—C3—C4	4.6 (3)
C2—S1—C1—N2	−1.07 (13)	S1—C2—C3—C4	−176.16 (15)
N2—N3—C2—C3	178.87 (16)		

Symmetry code: (i) −*x*+2, −*y*+1, −*z*+1.

### 2-Amino-5-ethyl-1,3,4-thiadiazol-3-ium hemioxalate (I) Hydrogen-bond geometry (Å, º)

**Table:**

*D*—H···*A*	*D*—H	H···*A*	*D*···*A*	*D*—H···*A*
N1—H3*A*···O1	0.86	1.92	2.765 (2)	167
N1—H3*B*···O2^ii^	0.86	1.99	2.821 (2)	162
N2—H1···O2	0.92 (3)	1.78 (3)	2.6989 (19)	178 (2)
C3—H1*C*···O1^iii^	0.97	2.65	3.479 (2)	143

Symmetry codes: (ii) *x*−1, *y*, *z*; (iii) −*x*+3/2, *y*−1/2, −*z*+3/2.

### 4-(Dimethylamino)pyridin-1-ium 4-phenyl-5-sulfanylidene-4,5-dihydro-1,3,4-thiadiazole-2-thiolate (II) Crystal data

**Table:**

C_7_H_11_N_2_^+^·C_8_H_5_N_2_S_3_^−^	*F*(000) = 728
*M**_r_* = 348.50	*D*_x_ = 1.336 Mg m^−^^3^
Monoclinic, *P*2_1_/*n*	Cu *K*α radiation, λ = 1.54184 Å
*a* = 9.6422 (2) Å	Cell parameters from 6977 reflections
*b* = 17.1758 (3) Å	θ = 2.5–70.7°
*c* = 10.6080 (2) Å	µ = 3.92 mm^−^^1^
β = 99.546 (2)°	*T* = 293 K
*V* = 1732.49 (6) Å^3^	Needle, colourless
*Z* = 4	0.20 × 0.17 × 0.12 mm

### 4-(Dimethylamino)pyridin-1-ium 4-phenyl-5-sulfanylidene-4,5-dihydro-1,3,4-thiadiazole-2-thiolate (II) Data collection

**Table:**

XtaLAB Synergy, Single source at home/near, HyPix3000 diffractometer	2681 reflections with *I* > 2σ(*I*)
Detector resolution: 10.0000 pixels mm^-1^	*R*_int_ = 0.047
/ω scans	θ_max_ = 71.4°, θ_min_ = 5.0°
Absorption correction: multi-scan (CrysAlisPro; Rigaku OD, 2020)	*h* = −11→11
*T*_min_ = 0.123, *T*_max_ = 1.000	*k* = −18→21
16519 measured reflections	*l* = −13→12
3341 independent reflections	

### 4-(Dimethylamino)pyridin-1-ium 4-phenyl-5-sulfanylidene-4,5-dihydro-1,3,4-thiadiazole-2-thiolate (II) Refinement

**Table:**

Refinement on *F*^2^	Primary atom site location: dual
Least-squares matrix: full	Hydrogen site location: mixed
*R*[*F*^2^ > 2σ(*F*^2^)] = 0.047	H atoms treated by a mixture of independent and constrained refinement
*wR*(*F*^2^) = 0.142	*w* = 1/[σ^2^(*F*_o_^2^) + (0.070*P*)^2^ + 0.4264*P*] where *P* = (*F*_o_^2^ + 2*F*_c_^2^)/3
*S* = 1.09	(Δ/σ)_max_ = 0.001
3341 reflections	Δρ_max_ = 0.43 e Å^−^^3^
234 parameters	Δρ_min_ = −0.48 e Å^−^^3^
0 restraints	

### 4-(Dimethylamino)pyridin-1-ium 4-phenyl-5-sulfanylidene-4,5-dihydro-1,3,4-thiadiazole-2-thiolate (II) Special details

**Table:**

Geometry. All esds (except the esd in the dihedral angle between two l.s. planes) are estimated using the full covariance matrix. The cell esds are taken into account individually in the estimation of esds in distances, angles and torsion angles; correlations between esds in cell parameters are only used when they are defined by crystal symmetry. An approximate (isotropic) treatment of cell esds is used for estimating esds involving l.s. planes.

### 4-(Dimethylamino)pyridin-1-ium 4-phenyl-5-sulfanylidene-4,5-dihydro-1,3,4-thiadiazole-2-thiolate (II) Fractional atomic coordinates and isotropic or equivalent isotropic displacement parameters (Å^2^)

**Table:**

	*x*	*y*	*z*	*U*_iso_*/*U*_eq_	
S1	0.98676 (7)	0.65986 (4)	0.81645 (7)	0.0706 (2)	
S2	1.24460 (7)	0.58869 (4)	0.73915 (8)	0.0745 (2)	
S3	0.68531 (7)	0.63843 (5)	0.85224 (7)	0.0758 (2)	
N2	0.9940 (2)	0.52129 (11)	0.75216 (19)	0.0578 (5)	
N1	0.8566 (2)	0.52989 (13)	0.7759 (2)	0.0655 (5)	
N4	0.3738 (3)	0.62373 (15)	0.1821 (2)	0.0773 (6)	
N3	0.5545 (3)	0.61788 (18)	0.5594 (3)	0.0861 (7)	
C3	1.0350 (3)	0.44426 (13)	0.7193 (2)	0.0592 (6)	
C11	0.4299 (3)	0.62077 (14)	0.3046 (2)	0.0597 (6)	
C2	1.0800 (3)	0.58276 (14)	0.7660 (2)	0.0581 (6)	
C1	0.8367 (3)	0.60132 (16)	0.8129 (2)	0.0601 (6)	
C12	0.3763 (3)	0.66388 (16)	0.3991 (3)	0.0679 (7)	
C4	1.1299 (3)	0.40347 (16)	0.8063 (3)	0.0704 (7)	
C8	0.9777 (3)	0.41273 (15)	0.6033 (3)	0.0706 (7)	
C10	0.5502 (4)	0.57518 (18)	0.3494 (3)	0.0783 (8)	
C13	0.4416 (4)	0.66102 (18)	0.5222 (3)	0.0781 (8)	
C5	1.1668 (4)	0.32861 (18)	0.7750 (4)	0.0859 (9)	
C7	1.0156 (4)	0.33772 (18)	0.5741 (4)	0.0852 (9)	
C6	1.1092 (4)	0.29664 (18)	0.6601 (4)	0.0890 (10)	
C9	0.6068 (4)	0.5752 (2)	0.4736 (4)	0.0905 (10)	
C14	0.2475 (4)	0.6678 (3)	0.1364 (4)	0.1091 (13)	
H14A	0.224003	0.662573	0.045294	0.164*	
H14B	0.171551	0.648329	0.175580	0.164*	
H14C	0.263244	0.721689	0.158169	0.164*	
C15	0.4305 (5)	0.5763 (2)	0.0877 (4)	0.1113 (12)*	
H15A	0.377399	0.585714	0.004314	0.167*	
H15B	0.527201	0.589807	0.088255	0.167*	
H15C	0.424084	0.522178	0.108847	0.167*	
H8	0.910 (3)	0.4407 (17)	0.543 (3)	0.076 (8)*	
H12	0.302 (3)	0.6942 (18)	0.379 (3)	0.073 (8)*	
H4	1.168 (3)	0.4241 (18)	0.883 (3)	0.083 (10)*	
H5	1.235 (4)	0.300 (2)	0.840 (3)	0.104 (11)*	
H6	1.130 (4)	0.247 (2)	0.638 (4)	0.107 (11)*	
H7	0.976 (4)	0.316 (2)	0.490 (4)	0.094 (10)*	
H13	0.411 (4)	0.689 (2)	0.582 (3)	0.094 (11)*	
H11	0.594 (4)	0.544 (2)	0.297 (4)	0.103 (11)*	
H9	0.689 (4)	0.549 (2)	0.509 (4)	0.112 (12)*	
H3	0.596 (5)	0.619 (3)	0.654 (5)	0.141 (15)*	

### 4-(Dimethylamino)pyridin-1-ium 4-phenyl-5-sulfanylidene-4,5-dihydro-1,3,4-thiadiazole-2-thiolate (II) Atomic displacement parameters (Å^2^)

**Table:**

	*U*^11^	*U*^22^	*U*^33^	*U*^12^	*U*^13^	*U*^23^
S1	0.0638 (4)	0.0629 (4)	0.0867 (5)	0.0005 (3)	0.0171 (3)	−0.0164 (3)
S2	0.0614 (4)	0.0636 (4)	0.1027 (6)	−0.0064 (3)	0.0262 (4)	−0.0117 (3)
S3	0.0628 (4)	0.0961 (5)	0.0710 (4)	0.0094 (3)	0.0188 (3)	−0.0041 (3)
N2	0.0532 (10)	0.0552 (10)	0.0656 (12)	−0.0011 (8)	0.0111 (9)	−0.0017 (9)
N1	0.0545 (11)	0.0690 (13)	0.0740 (13)	−0.0029 (9)	0.0140 (10)	−0.0001 (10)
N4	0.0913 (17)	0.0815 (16)	0.0605 (13)	−0.0169 (13)	0.0170 (12)	−0.0015 (11)
N3	0.0877 (18)	0.0956 (19)	0.0729 (17)	−0.0155 (15)	0.0071 (14)	0.0087 (14)
C3	0.0622 (13)	0.0476 (12)	0.0691 (15)	−0.0027 (10)	0.0144 (11)	0.0033 (10)
C11	0.0661 (14)	0.0540 (12)	0.0633 (14)	−0.0110 (10)	0.0235 (11)	0.0004 (10)
C2	0.0612 (13)	0.0561 (13)	0.0570 (13)	−0.0007 (10)	0.0093 (10)	−0.0025 (10)
C1	0.0578 (13)	0.0724 (15)	0.0501 (12)	0.0025 (11)	0.0086 (10)	0.0009 (11)
C12	0.0692 (16)	0.0629 (15)	0.0768 (18)	0.0049 (12)	0.0272 (14)	0.0039 (12)
C4	0.0733 (17)	0.0611 (15)	0.0756 (18)	−0.0016 (12)	0.0087 (14)	0.0102 (13)
C8	0.0805 (18)	0.0574 (14)	0.0718 (17)	−0.0006 (12)	0.0066 (14)	−0.0005 (12)
C10	0.0807 (19)	0.0698 (17)	0.091 (2)	0.0110 (14)	0.0347 (17)	−0.0001 (15)
C13	0.100 (2)	0.0739 (18)	0.0672 (18)	−0.0099 (16)	0.0321 (17)	−0.0075 (14)
C5	0.086 (2)	0.0613 (17)	0.110 (3)	0.0093 (14)	0.0164 (19)	0.0230 (17)
C7	0.112 (3)	0.0602 (16)	0.086 (2)	−0.0047 (16)	0.0224 (19)	−0.0119 (15)
C6	0.100 (2)	0.0514 (15)	0.121 (3)	0.0044 (15)	0.035 (2)	0.0034 (17)
C9	0.076 (2)	0.098 (2)	0.098 (3)	0.0119 (17)	0.0139 (18)	0.0213 (19)
C14	0.096 (3)	0.135 (3)	0.088 (2)	−0.019 (2)	−0.009 (2)	0.029 (2)

### 4-(Dimethylamino)pyridin-1-ium 4-phenyl-5-sulfanylidene-4,5-dihydro-1,3,4-thiadiazole-2-thiolate (II) Geometric parameters (Å, º)

**Table:**

S1—C2	1.735 (2)	C4—C5	1.389 (4)
S1—C1	1.757 (3)	C4—H4	0.91 (3)
S2—C2	1.661 (3)	C8—C7	1.388 (4)
S3—C1	1.707 (3)	C8—H8	0.96 (3)
N2—C2	1.336 (3)	C10—C9	1.340 (5)
N2—N1	1.397 (3)	C10—H11	0.93 (4)
N2—C3	1.440 (3)	C13—H13	0.89 (3)
N1—C1	1.312 (3)	C5—C6	1.368 (5)
N4—C11	1.323 (4)	C5—H5	1.00 (4)
N4—C14	1.447 (5)	C7—C6	1.368 (5)
N4—C15	1.465 (5)	C7—H7	0.98 (4)
N3—C13	1.322 (5)	C6—H6	0.92 (4)
N3—C9	1.331 (5)	C9—H9	0.94 (4)
N3—H3	1.02 (5)	C14—H14A	0.9600
C3—C8	1.374 (4)	C14—H14B	0.9600
C3—C4	1.378 (4)	C14—H14C	0.9600
C11—C12	1.411 (4)	C15—H15A	0.9600
C11—C10	1.415 (4)	C15—H15B	0.9600
C12—C13	1.353 (5)	C15—H15C	0.9600
C12—H12	0.88 (3)		
			
C2—S1—C1	91.37 (12)	C7—C8—H8	119.5 (17)
C2—N2—N1	119.1 (2)	C9—C10—C11	120.5 (3)
C2—N2—C3	124.2 (2)	C9—C10—H11	116 (2)
N1—N2—C3	116.58 (19)	C11—C10—H11	124 (2)
C1—N1—N2	110.1 (2)	N3—C13—C12	122.4 (3)
C11—N4—C14	122.2 (3)	N3—C13—H13	117 (2)
C11—N4—C15	120.8 (3)	C12—C13—H13	121 (2)
C14—N4—C15	116.8 (3)	C6—C5—C4	120.2 (3)
C13—N3—C9	119.5 (3)	C6—C5—H5	123 (2)
C13—N3—H3	117 (3)	C4—C5—H5	117 (2)
C9—N3—H3	124 (3)	C6—C7—C8	120.0 (3)
C8—C3—C4	121.6 (2)	C6—C7—H7	121 (2)
C8—C3—N2	119.6 (2)	C8—C7—H7	119 (2)
C4—C3—N2	118.9 (2)	C7—C6—C5	120.8 (3)
N4—C11—C12	122.6 (3)	C7—C6—H6	117 (2)
N4—C11—C10	122.0 (3)	C5—C6—H6	122 (2)
C12—C11—C10	115.4 (3)	N3—C9—C10	122.2 (3)
N2—C2—S2	128.55 (19)	N3—C9—H9	113 (2)
N2—C2—S1	107.04 (18)	C10—C9—H9	125 (2)
S2—C2—S1	124.40 (15)	N4—C14—H14A	109.5
N1—C1—S3	126.6 (2)	N4—C14—H14B	109.5
N1—C1—S1	112.37 (18)	H14A—C14—H14B	109.5
S3—C1—S1	121.04 (16)	N4—C14—H14C	109.5
C13—C12—C11	120.0 (3)	H14A—C14—H14C	109.5
C13—C12—H12	119 (2)	H14B—C14—H14C	109.5
C11—C12—H12	121 (2)	N4—C15—H15A	109.5
C3—C4—C5	118.6 (3)	N4—C15—H15B	109.5
C3—C4—H4	122 (2)	H15A—C15—H15B	109.5
C5—C4—H4	120 (2)	N4—C15—H15C	109.5
C3—C8—C7	118.9 (3)	H15A—C15—H15C	109.5
C3—C8—H8	121.6 (17)	H15B—C15—H15C	109.5
			
C2—N2—N1—C1	−1.6 (3)	C2—S1—C1—N1	−0.3 (2)
C3—N2—N1—C1	175.7 (2)	C2—S1—C1—S3	−179.33 (16)
C2—N2—C3—C8	−113.1 (3)	N4—C11—C12—C13	−177.2 (3)
N1—N2—C3—C8	69.7 (3)	C10—C11—C12—C13	1.4 (4)
C2—N2—C3—C4	67.7 (3)	C8—C3—C4—C5	−0.4 (4)
N1—N2—C3—C4	−109.4 (3)	N2—C3—C4—C5	178.7 (3)
C14—N4—C11—C12	−4.0 (4)	C4—C3—C8—C7	0.6 (4)
C15—N4—C11—C12	−178.3 (3)	N2—C3—C8—C7	−178.5 (3)
C14—N4—C11—C10	177.5 (3)	N4—C11—C10—C9	178.1 (3)
C15—N4—C11—C10	3.2 (4)	C12—C11—C10—C9	−0.5 (4)
N1—N2—C2—S2	−177.69 (18)	C9—N3—C13—C12	−0.2 (5)
C3—N2—C2—S2	5.2 (4)	C11—C12—C13—N3	−1.1 (5)
N1—N2—C2—S1	1.3 (3)	C3—C4—C5—C6	−0.2 (5)
C3—N2—C2—S1	−175.83 (19)	C3—C8—C7—C6	−0.3 (5)
C1—S1—C2—N2	−0.50 (18)	C8—C7—C6—C5	−0.2 (5)
C1—S1—C2—S2	178.52 (17)	C4—C5—C6—C7	0.5 (5)
N2—N1—C1—S3	179.97 (17)	C13—N3—C9—C10	1.2 (5)
N2—N1—C1—S1	1.0 (3)	C11—C10—C9—N3	−0.7 (5)

### 4-(Dimethylamino)pyridin-1-ium 4-phenyl-5-sulfanylidene-4,5-dihydro-1,3,4-thiadiazole-2-thiolate (II) Hydrogen-bond geometry (Å, º)

**Table:**

*D*—H···*A*	*D*—H	H···*A*	*D*···*A*	*D*—H···*A*
N3—H3···S3	1.02 (5)	2.16 (5)	3.173 (3)	173 (4)
